# Methotrexate Modulates Polymorphonuclear Neutrophil Function Through Reduced Chemotaxis and Increased LOX‐1 Expression

**DOI:** 10.1002/iid3.70537

**Published:** 2026-10-06

**Authors:** Viktor Wieke, Richard Felix Kraus, Michael Andreas Gruber, Martin Kieninger

**Affiliations:** ^1^ Department of Anesthesiology University Hospital Regensburg Regensburg Germany

**Keywords:** chemotaxis, LOX‐1, methotrexate, PMN function, surface epitopes

## Abstract

**Background:**

Methotrexate (MTX) is commonly used in patients with autoimmune disorders with the therapeutic objective of inducing immunosuppression predominantly through the modulation of T‐lymphocyte activity. Additional studies suggest that the regulation of polymorphonuclear neutrophils (PMNs) may also play a significant role in disease modification. However, the molecular mechanism underlying this effect remains incompletely understood. Moreover, most studies investigating the interaction between MTX and PMNs have relied on centrifugation for cell isolation, a method meanwhile known to alter PMN behavior and activation status itself.

**Objectives:**

The aim of the study was to investigate the effects of MTX on PMN function—including reactive oxygen species (ROS) production, NETosis (Neutrophil Extracellular Traps), and migration—using live‐cell imaging, as well as surface epitopes and the respiratory burst assessed by flow cytometry. This comprehensive set of experiments examining the interaction between MTX and PMNs provides a previously unreported level of insight.

**Methods:**

PMNs from healthy donors were isolated using a sedimentation procedure with 10% Gelafundin and subsequently incubated with 0, 0.005, 0.5, or 5 mmol/L MTX for downstream experiments. Live‐cell imaging was used to assess neutrophil migration, NETosis, and ROS production, while flow cytometry was conducted to evaluate the activation markers CD11b, CD66b, and CD62L, as well as the surface marker LOX‐1 (lectin‐type oxidized LDL receptor‐1) and ROS production.

**Results:**

Live‐cell imaging demonstrated a significant inhibition of chemotactic PMN migration at all MTX concentrations compared to controls. In contrast, neither ROS production nor NETosis could be detected under the same conditions. Flow cytometric analysis revealed a significant upregulation of LOX‐1 surface expression, while the activation markers CD11b, CD62L, and CD66b remained unaltered. Furthermore, an increase in ROS production during the respiratory burst could not be detected.

**Conclusion:**

These results indicate that MTX reduces the chemotaxis of most naive PMNs. In addition, MTX appears to induce an upregulation of LOX‐1. Future studies should further investigate the impact of LOX‐1 on PMN function.

## 1 Introduction

PMNs represent the first line of cellular immune defense in the human body and are part of the innate immune system [1, 2]. Therefore, PMNs possess several defense mechanisms:

An essential function of PMNs is their ability to migrate. Migration is guided by both exogenous chemotactic factors, such as lipopolysaccharides and formyl‐methionyl‐leucyl‐phenylalanine (fMLP), as well as endogenous cytokines, including tumor necrosis factor alpha, interleukin‐1, and interleukin‐8 [2, 3]. Another key antimicrobial mechanism is the so‐called respiratory burst, characterized by the formation of reactive oxygen species. The enzyme NADPH oxidase serves as a central catalyst in this process, generating reactive intermediates [4, 5]. Another mechanism by which pathogens can be targeted involves neutrophil extracellular traps—networks of extracellular chromatin fibers that immobilize bacteria [6, 7].

These mechanisms are regulated, among other factors, by surface markers of PMNs. CD11b, a component of the integrin Mac‐1, is upregulated upon activation and plays a crucial role in the migration of PMNs into inflamed tissue [8, 9]. CD66b is considered a hallmark marker of PMN activation. It modulates the activity of CD11 and CD18 and thus contributes to adhesion and migration processes [10, 11]. CD62L (l‐Selectin) serves as a key component in diapedesis into the affected site [12].

In several autoimmune conditions, including rheumatoid arthritis (RA) and psoriasis vulgaris, these defense mechanisms become uncontrolled and ultimately damage host tissues [13]. Until the early 2000s, research in this field primarily focused on the adaptive immune system, particularly on T and B lymphocytes. In recent years, however, attention has increasingly shifted toward the innate immune system. Especially, PMNs and their capacity for ROS generation and NETosis play a central role in pathogenesis. However, these conditions remain challenging to treat for clinicians. One of the most important therapeutic options in RA is methotrexate [14, 15].

MTX has been part of the standard therapeutic repertoire in rheumatology since the 1980s. It is a competitive inhibitor of dihydrofolate reductase that exerts its primary effects through inhibition of DNA synthesis by catalyzing the conversion of dihydrofolate to its active form, tetrahydrofolate [16, 17]. However, this mechanism cannot fully explain the drug's effects, since methotrexate also influences cells with low proliferative rates.

Additionally, recent studies have also reported elevated LOX‐1 levels in patients with RA. LOX‐1 is a receptor for oxidized low‐density lipoprotein that is primarily expressed on endothelial cells and macrophages. More recent studies have demonstrated that LOX‐1 is also expressed on PMNs, where it appears to exert an immunomodulatory and potentially even immunosuppressive function [18]. LOX‐1 has therefore been proposed as a potential biomarker reflecting disease activity in RA [19, 20]. The present study also investigates the effect of MTX on the expression of LOX‐1, as a potential confounder of the LOX‐1 upregulation.

However, many of these data are based on *in vitro* experiments in which PMNs are isolated by centrifugation. Recent findings from our research group, however, indicate that this centrifugation step markedly impairs PMN function, such as migration and antigen expression. The underlying mechanism appears to involve the intracellular release of granule contents during centrifugation. These findings challenge previous results and provide a foundation for further research. Therefore, the experiments conducted in this thesis were performed using a separation method in which centrifugation could be deliberately omitted [21, 22, 23].

The aim of this study was to gain insight into how methotrexate modulates and inhibits PMN function, and to investigate the potential role of LOX‐1. To investigate this, we employed a complementary experimental approach combining live‐cell imaging with flow‐cytometric analyses.

## 2 Material and Methods

### 2.1 Blood Collection and PMN Isolation

In accordance with the Declaration of Helsinki (vote of the local ethics committee of the University of Regensburg [file number: 18‐1210‐101]) informed consent was obtained and whole blood was drawn from healthy volunteer donors from the cubital vein using butterfly needles (Sarstedt AG & Co. KG, Nümbrecht, Germany) and collected in lithium heparin Monovettes (Sarstedt AG & Co. KG, Nümbrecht, Germany). The blood was then mixed with 10% Gelafundin (Serumwerk Bernburg AG, Bernburg, Germany), swirled, and left to stand until a supernatant formed during erythrocyte sedimentation. The leukocyte‐rich plasma could then be collected after 30 min at room temperature.

Autologous plasma was obtained by centrifuging (Heraeus Biofuge stratos, Heraeus Deutschland GmbH & Co. KG, Hanau, Germany) blood collected in a serum tube at 1180 *× g* for 10 min at 21°C.

### 2.2 Setup of the Chemotaxis Assay

Chemotaxis was assessed using 3D µ‐Chemotaxis slides from Ibidi (µ‐Slide VI 0.1, ibidi GmbH, Martinsried, Germany). Each slide consists of three independent chamber systems, each composed of two reservoirs connected by a central channel. The chambers were filled through small openings at the edges, which were subsequently sealed with plugs.

The chambers were filled with a mixture of type I collagen matrix (PureCol Bovine Collagen Solution Type 1, 3 mg/mL, Advanced BioMatix, Carlsbad, USA) and RPMI (Pan Biotech Ltd., Aidenbach, Germany) containing either 0, 0.5, or 5 mmol/L MTX. 1.8 × 10^6^ PMNs/mL were added to one side of the chamber containing 50% autologous plasma. To establish a chemotactic gradient, the opposite reservoir was filled with the chemoattractant fMLP (10 nM, Sigma‐Aldrich Chemie GmbH, Steinheim, Deutschland) or IL‐8 (100 ng/mL, Sigma‐Aldrich Chemie GmbH, Steinheim, Deutschland) dissolved in 50% autologous plasma, as explained in an earlier publication of our research group [24].

### 2.3 Live Cell Imaging

The microscope used in this study consists of an inverted microscope system Leica DMi8 (Leica Mikroskopie & Systeme GmbH, Wetzlar, Germany), equipped with an HC PL FL L 20x/0.40 CORR PH1 objective (Leica Mikroskopie & Systeme GmbH, Wetzlar, Germany) and a CoolLED pE‐4000 illumination source (CoolLED Ltd, Andover, United Kingdom).

To replicate physiological conditions within the experimental setup, the chamber systems were fixed on the microscope stage inside a climate chamber (ibidi Heating System, Universal Fit, for 1 Chamber, Ibidi, Martinsried, Germany). This climate chamber maintained constant environmental parameters (37°C, 5% CO_2,_ and 50% air humidity). Subsequently, the positions of the channels were defined and brought into focus in phase contrast using the software Leica Application Suite X version 3.4.2.18368 (Leica Microsystems CMS GmbH, Wetzlar, Germany). The observation period lasted 21.7 h, during which the microscope camera captured one image of each channel every 30 s, resulting in a total of 2600 images.

ROS production was detected using 1 µM of the fluorescent dye Dihydrorhodamine‐123 (DHR, Life Technologies, Eugene, Oregon, USA), and NETosis was assessed using 0.083 µg/mL of the dye 4′,6‐Diamidino‐2‐phenylindole (DAPI, Sigma‐Aldrich Chemie GmbH, Steinheim, Deutschland), as previously demonstrated in our group's earlier studies [25].

Figure 1 illustrates the experimental setup.

**Figure 1:**
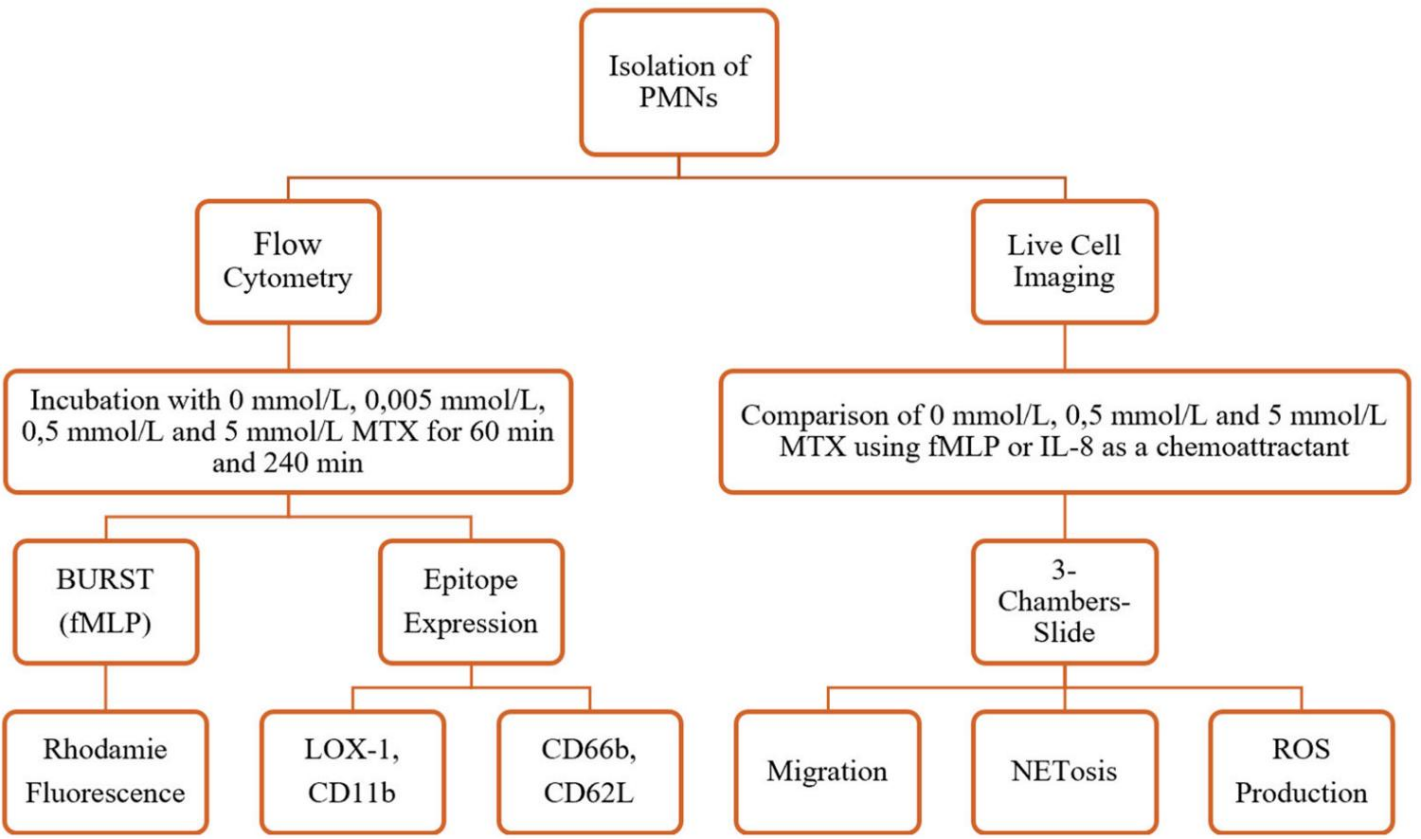
Schematic of the experimental design.

### 2.4 Evaluation of Migration

Image analysis was performed using the software Imaris 9.0.2 (Bitplane AG, Belfast, UK). Each individual cell was identified within a coordinate system. Cells were tracked for 60 consecutive images, corresponding to a time span of 30 min. During the first 180 min, cells were continuously analyzed in 30 min periods, and subsequently only every 250 min. Erroneously detected artifacts and short tracks were excluded using the filters Track Length < 25 µm and Track Duration < 900 ms. To ensure better comparability, only the PMNs with the 50 longest track lengths were selected for analysis in each experimental setup. Also, cell analysis commenced only from minute 61 onward. The following parameters (see Table 1) were calculated by Imaris:

**Table 1:** Explanation of the analyzed parameters in Imaris.

Parameter	Description
Track length (TL)	Total distance traveled by a PMN [µm]
Track displacement X (TDX)	Distance traveled along the X‐axis, corresponding to the chemotactic gradient (movement toward the chemokine) [µm]
Track displacement Y (TDY)	Distance traveled along the Y‐axis [µm]
Track displacement length (TDL)	Euclidic distance between the start and end points of a track [µm]
Track straightness (TS)	Ratio of Track Displacement Length to Track Length; indicates the linearity of cell migration
Track duration (TD)	Duration of a track [s]

### 2.5 Evaluation of the Flow Cytometry Experiments

For the flow cytometry experiments, both the oxidative burst and the surface epitope measurement were performed.

#### 2.5.1 Measurement of the Respiratory Burst

At the beginning of the experiment, the cells were treated with methotrexate and incubated in a water bath for 60 min and 240 min at 37°C, respectively. Four different concentrations were prepared: 0, 0.005, 0.5, and 5 mmol/L methotrexate. The sample containing 0 mmol/L served as the control.

For the burst assay, three separate tubes were prepared in duplicate for each of the four different concentrations.

First, 1 mL of PBS was mixed with 20 µL of leukocyte‐rich plasma and 10 µL each of DHR and SNARF (Thermo Fisher Scientific, Waltham, USA). Additionally, 10 µL of TNF‐α (PeproTech Germany, Hamburg, Germany) was added to each designated tube. All samples were then incubated in a water bath at 37°C for 10 min.

Subsequently, 10 µL of fMLP (Sigma‐Aldrich Chemie GmbH, Steinheim, Germany) was added to the samples containing TNF‐α. For the positive control, 10 µL of PMA (Sigma‐Aldrich, Steinheim, Germany) was added to the preparations lacking TNF‐α and fMLP (representative images of the positive control are shown in Figure 1 Supplement). All samples were then incubated in a water bath at 37°C for 20 min, followed by cooling at 4°C for at least 5 min.

Abrupt cooling stopped ROS formation, ensuring that the amount of rhodamine‐123 remained constant. As a final step, 10 µL of propidium iodide (Thermo Fisher Scientific, Waltham, USA), a substrate that detects nonvital cells, was added, and the measurement was initiated.

#### 2.5.2 Analysis of Surface Epitope Density

To detect surface markers, fluorophore‐conjugated antibodies from BioLegend (San Diego, USA) were used. For each MTX concentration, three samples were prepared in duplicate. One sample served as a blank control, the second was used for the simultaneous detection of CD11b‐PE, CD62L‐FITC, and CD66b‐APC, and the third for the detection of LOX‐1‐APC.

20 µL of the leukocyte‐rich plasma (see section 2.1) was mixed with 30 µL of cold PBS containing Ca^2+^/Mg^2+^ (Dulbecco's Phosphate Buffered Saline, Sigma‐Aldrich Chemie GmbH, Steinheim, Germany), followed by the addition of 5 µL of fluorophore‐conjugated antibodies. After incubation for 15 min at 4°C, the samples were washed with 2 mL of cold PBS (including Ca^2+^/Mg^2+^) and centrifuged at 425 × *g* for 5 min at 4°C. The supernatant was then discarded, the pellet resuspended in 250 µL of PBS (including Ca^2+^/Mg^2+^), and the measurement was initiated.

#### 2.5.3 Analysis of Flow Cytometry Results

Respiratory burst and antibody adhesion were measured using a FACSCalibur flow cytometer (Becton Dickinson, Franklin Lakes, USA). Data analysis was performed using FlowJo software, version X.0.7 (FlowJo LLC, Ashland, USA).

PMNs were initially gated according to their forward and side scatter properties. Propidium iodide–positive dead cells were excluded, and ROS production was then quantified.

To gain mean fluorescence intensities, gated PMNs were analyzed by plotting fluorescence intensity against side scatter parameters (see Figure 2). The results were reported in artificial fluorescence units [AFU].

**Figure 2:**
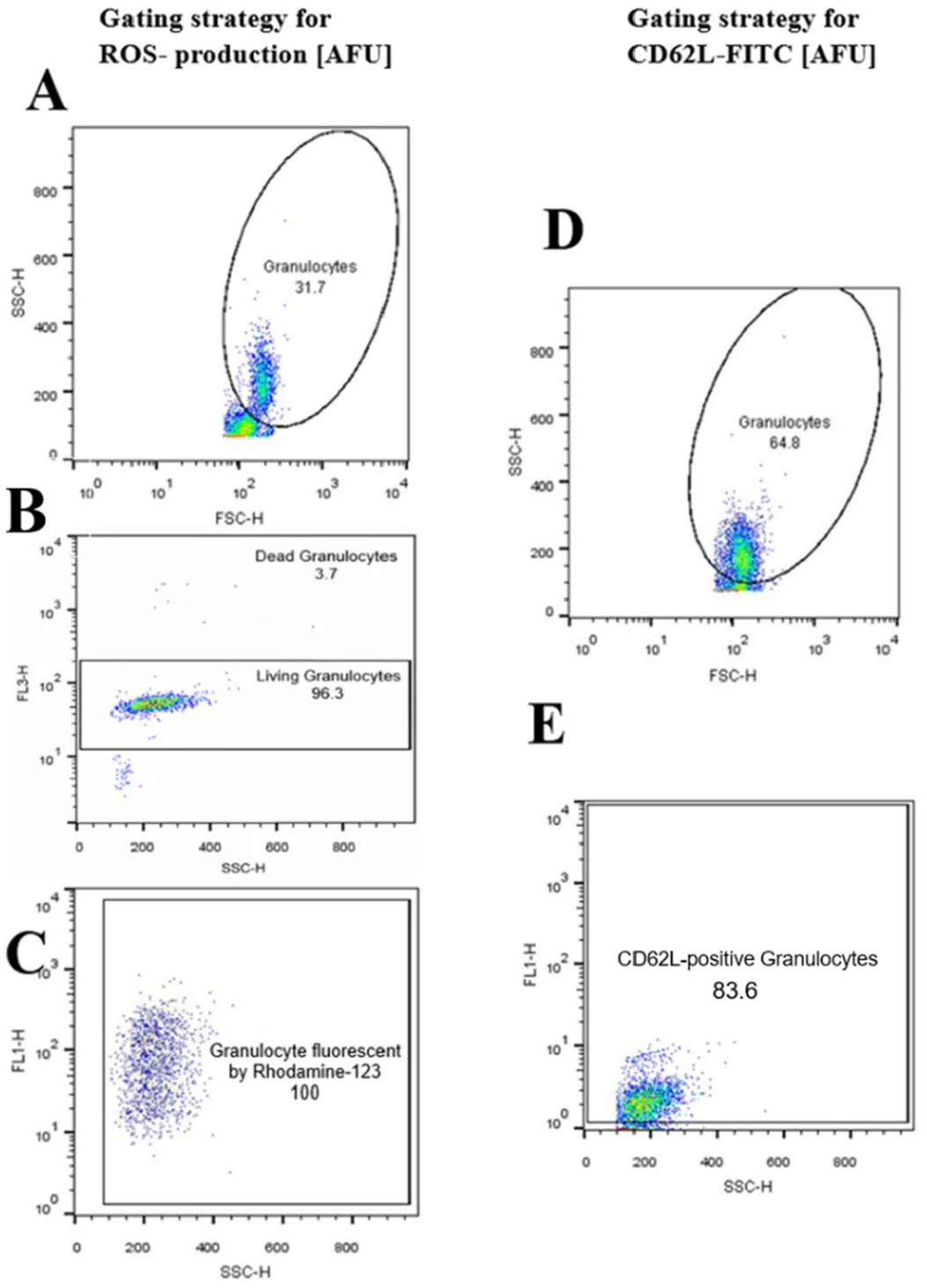
Example graphs generated using FlowJo software. Graphs (A–C) illustrate the workflow used to assess ROS production. Numbers indicate the percentage [%] of analyzed cells. Axis labels: SSC‐H: side scatter height; FSC‐H: forward scatter height; FL1‐H: Rhodamine‐123 or CD62L‐FITC response in [AFU], FL3‐H: Propidiumiodide response in [AFU]. (A) PMNs were distinguished from other cells based on size and granularity. (B) Living cells were separated from dead cells using PI response. (C) Rhodamine‐123 fluorescence signal. (D, E) Visualization of the CD62L‐FITC fluorescence response after isolation of PMNs.

### 2.6 Statistics

Statistical analyses were performed using IBM SPSS Statistics version 28. A significance level of *p* = 0.05 was set. Data were tested for normality using the Kolmogorov–Smirnov test. If the data were not normally distributed, non‐parametric tests were applied: the Mann–Whitney *U* test for two‐group comparisons and the Kruskal‐Wallis test for comparisons between more than two groups. For comparisons of normally distributed data, a *t*‐test was applied; when more than two groups were involved, analyses were conducted using ANOVA.

Flow cytometry data were presented as mean values with 95% confidence intervals when normally distributed and as boxplots with median values when distributional assumptions of normality were not met. Live‐cell imaging data were visualized using boxplots due to the absence of normal distribution.

In the plots, outliers are depicted as circles; these represent values exceeding 1.5 times the interquartile range (IQR) above the upper quartile or below the lower quartile. Extreme values are shown as asterisks and correspond to observations exceeding three times the IQR beyond the upper or lower quartile.

## 3 Results

In total, *n* = 17 blood samples were collected. Every single experiment was performed using PMNs from a different donor. Of these, *n* = 9 were male, and *n* = 8 were female. The age range of the participants was 22–59 years, and all subjects were healthy at the time of blood collection.

### 3.1 Analysis of the Chemotaxis Assay

In the statistical analysis of cell migration, tracks from *n* = 5 experiments were included for each MTX concentration (0, 0.5, and 5 mmol/L). As shown in Figure 3, significant differences of track lengths were observed between the groups. Each color represents a 30 min interval, starting at 61 min. These findings indicate that increasing MTX concentrations were associated with a reduction in track lengths.

**Figure 3:**
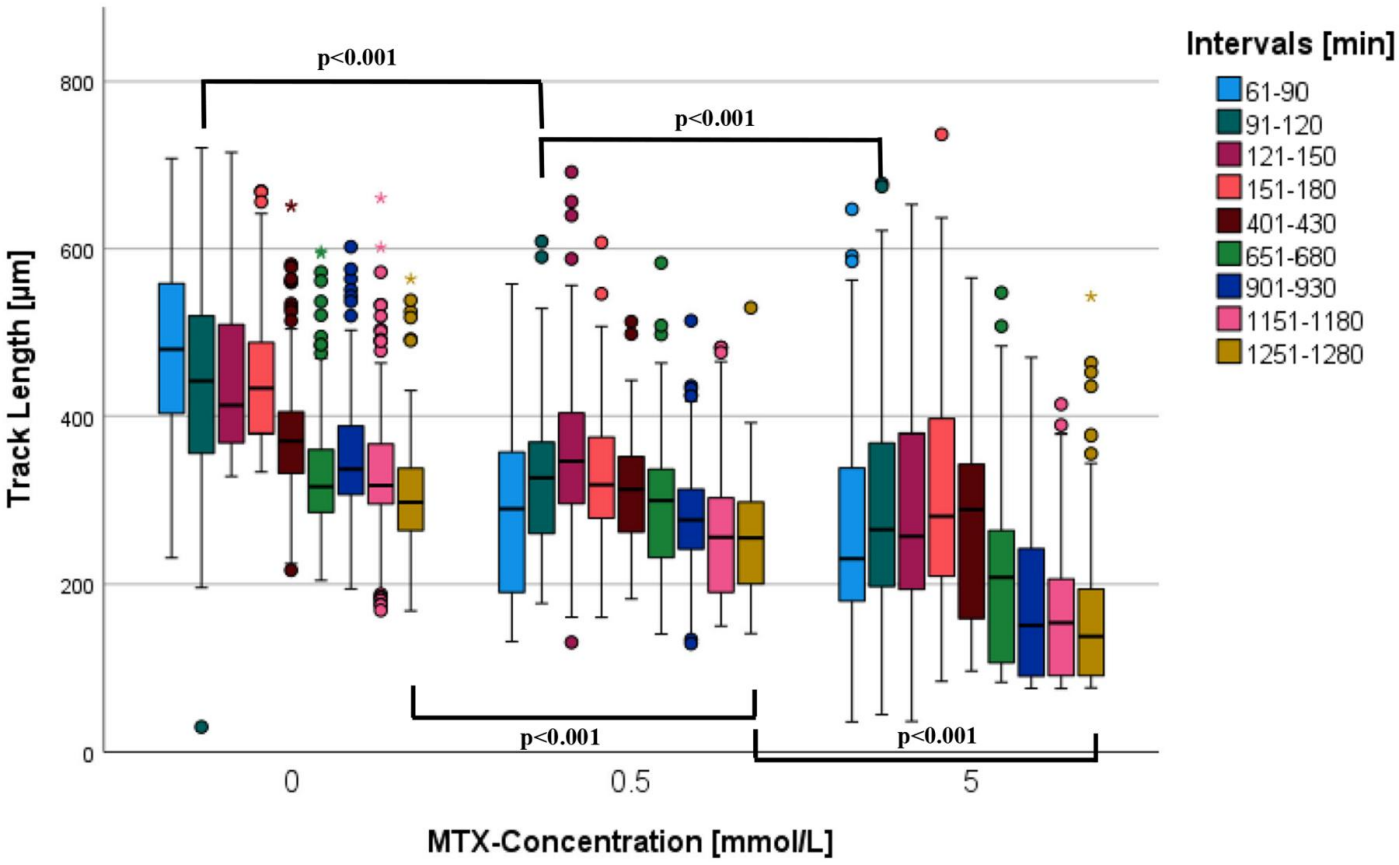
Track length in dependence on MTX concentration across 30‐min intervals using fMLP as a chemoattractant; circles denote outliers, whereas asterisks indicate extreme values.

Across the entire observation period, the median TL of all included tracks was 373.0 µm at 0 mmol/L MTX (*n* = 1891 tracks), with an IQR of 139.5. At an MTX concentration of 0.5 mmol/L (*n* = 1563 tracks), the median track length was 296.3 µm (IQR = 115.0), whereas at 5 mmol/L (*n* = 1696 tracks), the median track length decreased to 214.7 µm with an IQR of 171.6. This results in statistically significant differences (*p* < 0.001) between the three groups. Figure 4 illustrates this effect, showing a representative image obtained from the Imaris software.

**Figure 4:**
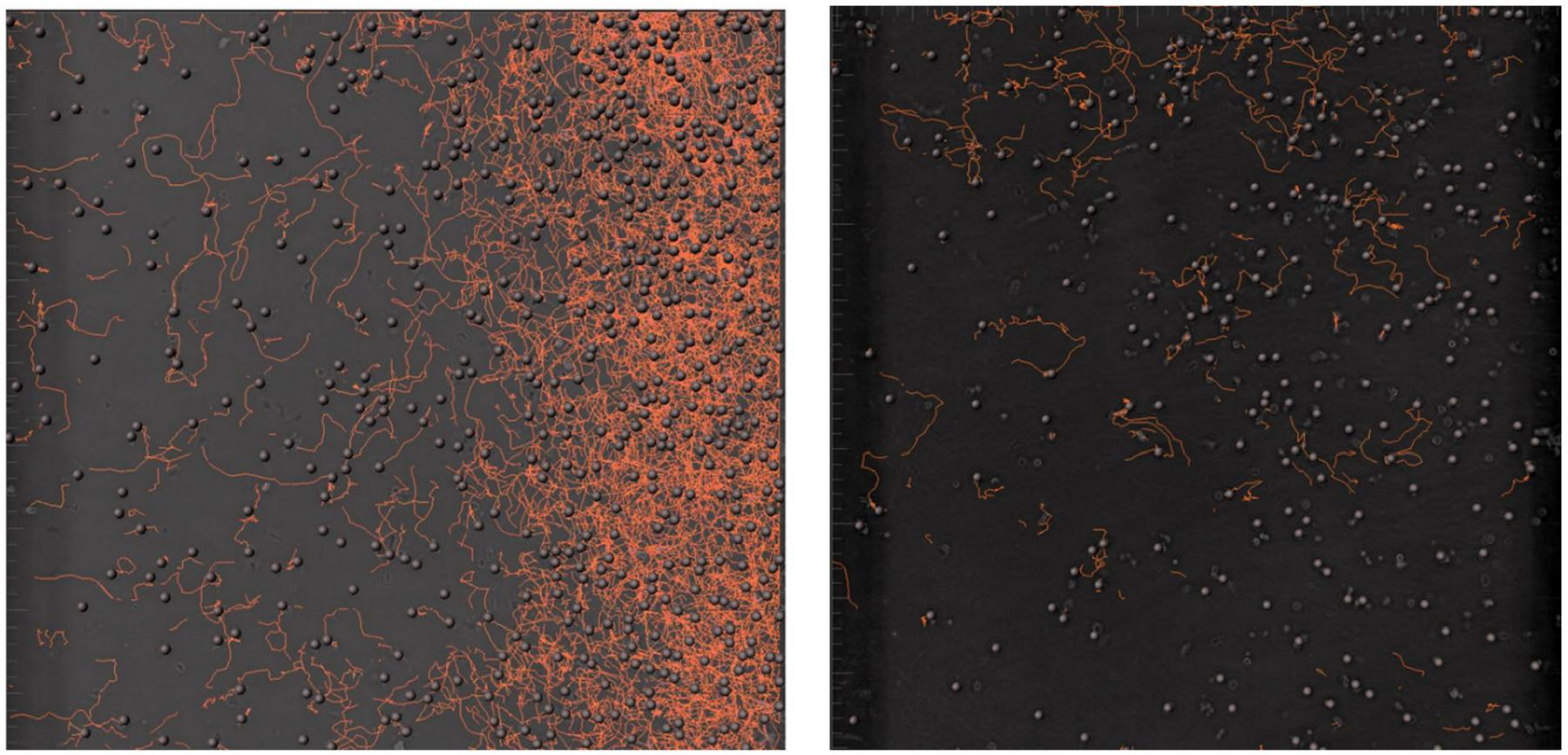
Screen shot of cell tracks by Imaris after 17 h of observation for MTX concentrations of 0 mmol/L (left) and 5 mmol/L (right).

Changes of TDX were observed dependent on MTX concentration (see Figure 5). The median TDX was 87.2 µm at 0 mmol/L MTX, including (*n* = 1891 tracks), 45.2 µm at 0.5 mmol/L (*n* = 1563 tracks), and 11.0 µm at 5 mmol/L (*n* = 1696 tracks), with all differences being statistically significant (*p* < 0.001).

**Figure 5:**
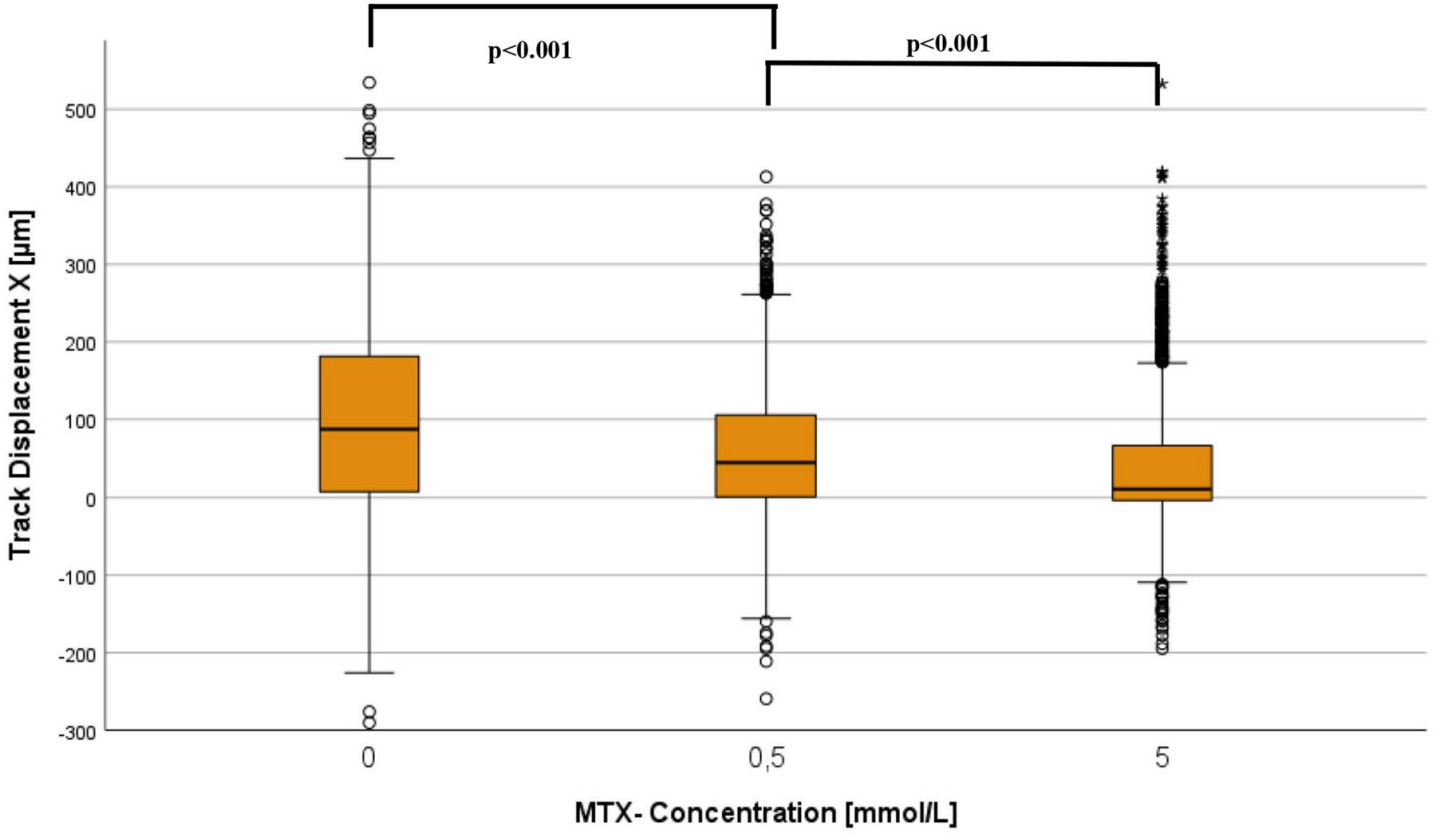
Median Track displacement X (µm) across all included observation intervals as a function of MTX concentration (mmol/L) and fMLP as a chemoattractant, circles denote outliers, whereas asterisks indicate extreme values.

In addition, the percentage decrease in TDX was compared with the relative decrease of track lengths (see Table 1 Supplement). The data show that TDX decreases proportionally more than the TL. At 0.5 mmol/L MTX, TL remains at 79.4% of the baseline value, whereas TDX is reduced to even 51.8%. This effect is even more pronounced at 5 mmol/L MTX, where TL corresponds to 57.6% of the baseline value, while TDX declines to merely 12.6%. All of these differences were statistically significant at *p* < 0.001.

The same experimental setup was repeated, again comparing 30 min intervals as described above, using IL ‐8 in place of fMLP as the chemokine (see Figure 6). At 0 mmol/L MTX (*n* = 2089 tracks), the median track length was 350.7 µm with an IQR of 109.1. At an MTX concentration of 0.5 mmol/L (*n* = 1594 tracks), the median track length was 326.0 µm (IQR = 97.7), while at 5 mmol/L (*n* = 2062 tracks) it decreased to 237.7 µm with an IQR of 80.5. The reduction in track length between all three groups was statistically significant (*p* < 0.001).

**Figure 6:**
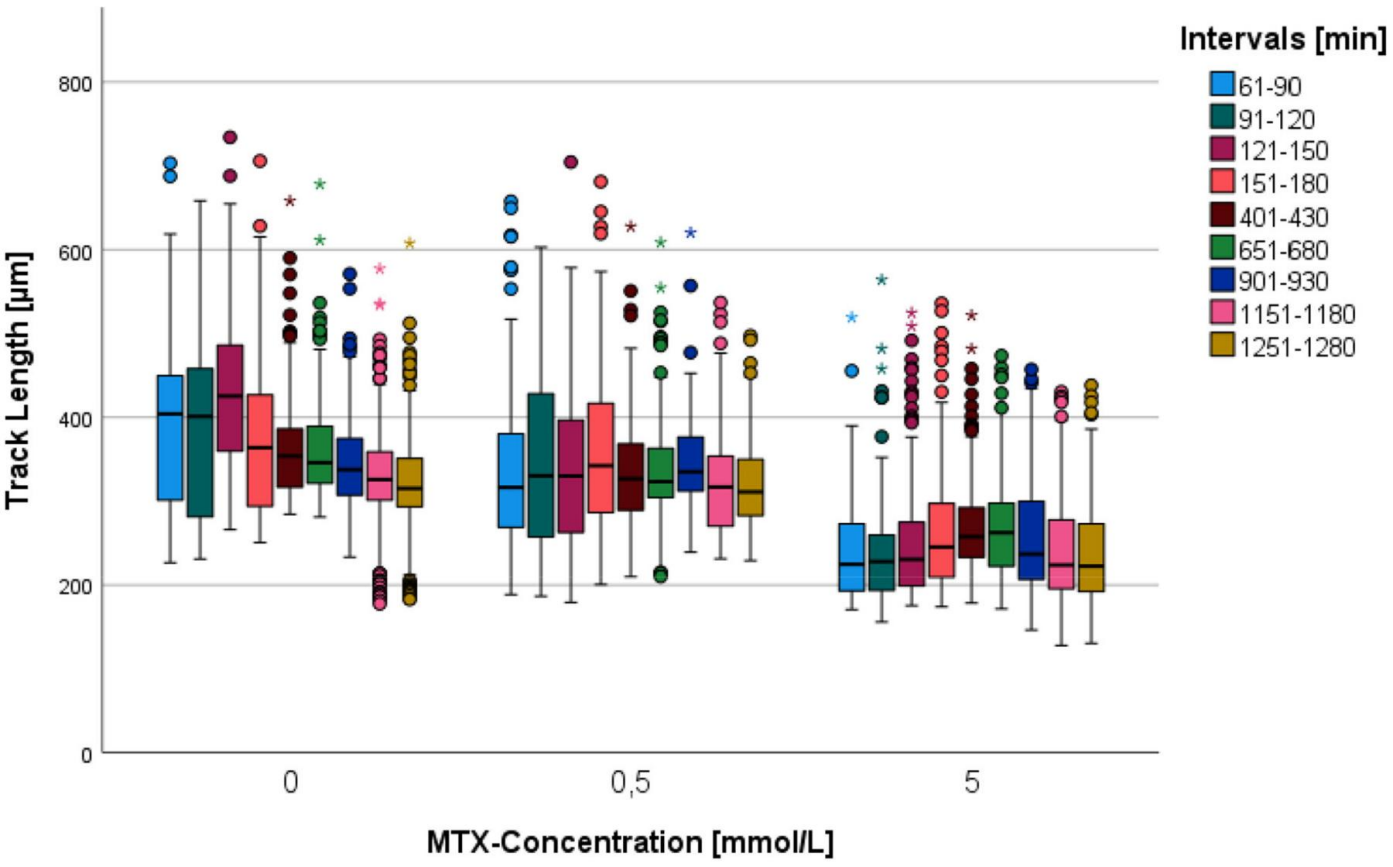
Track length (µm) as a function of MTX concentration (mmol/L) using interleukin‐8 as a chemoattractant, circles denote outliers, whereas asterisks indicate extreme values.

TDX was again compared as a function of MTX concentration, this time using IL‐8 as the chemoattractant shown in Figure 7. Each boxplot represents the median TDX across all included 30‐min intervals. The median TDX was 69.5 µm at 0 mmol/L MTX (*n* = 2089 tracks), 50.8 µm at 0.5 mmol/L (*n* = 1594 tracks), and 10.3 µm at 5 mmol/L (*n* = 2062 tracks), with all pairwise differences reaching statistical significance (*p* < 0.001).

**Figure 7:**
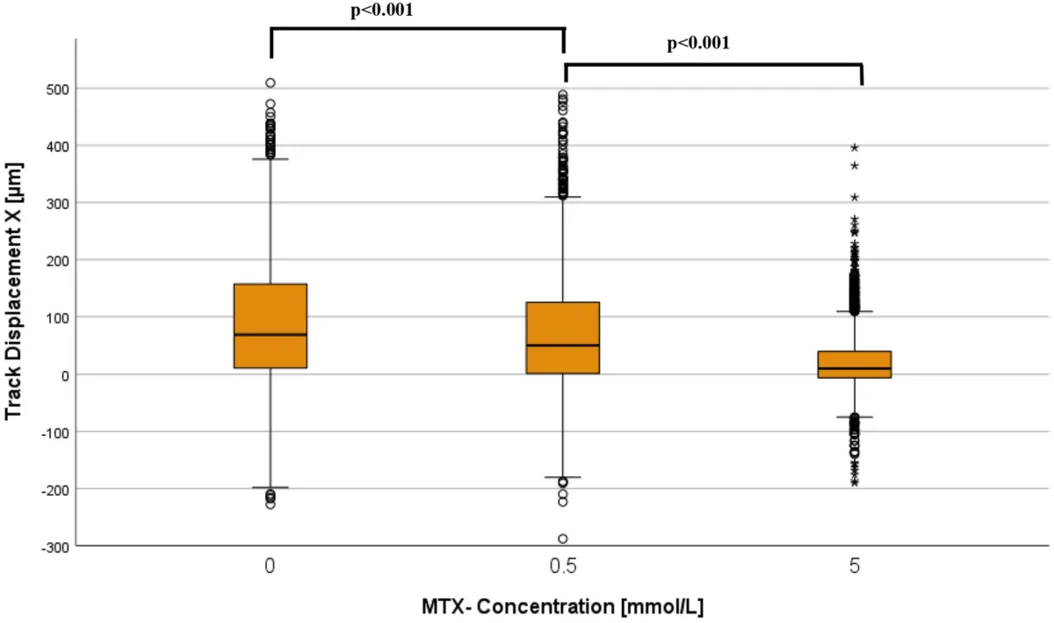
Track displacement X (µm) as a function of MTX concentration (mmol/L) and IL‐8 as a chemoattractant, circles denote outliers, whereas asterisks indicate extreme values.

Again, TDX‐ and thus chemotaxis‐ decreases proportionally more than the TL (see Table 2 Supplement). At 0.5 mmol/L MTX, TL remains at 93.0% of the baseline value, whereas TDX is reduced to 73.1%. This discrepancy becomes even more pronounced at 5 mmol/L MTX, where TL corresponds to 67.8% of baseline, while TDX decreases to only 14.8%. All of these differences were statistically significant (*p* < 0.001).

### 3.2 Evaluation of NETosis and ROS Generation Using Fluorescence Microscopy

No differences in PMN NETosis or ROS production were observed using Live‐cell imaging. The intensity analysis of the fluorescent dyes Rhodamine‐123 and DAPI showed no significant increase in fluorescence compared to the control.

### 3.3 Analysis of Flow Cytometry Data

#### 3.3.1 Analyses of Neutrophil Surface Epitopes

After testing for normal distribution, the expression levels of the surface markers CD11b, CD62L, and CD66b were analyzed. Data following a normal distribution are reported as means with corresponding 95% confidence intervals. Non‐normally distributed data are presented as medians and visualized using boxplots. Absolute values of the means or medians, along with the corresponding 95% confidence intervals or IQRs, are presented in Supplementary Table 3.

The evaluation of CD11b revealed no significant differences between groups (60 min: *p* = 0.344; 240 min: *p* = 0.861; Figure 8A,B).

**Figure 8:**
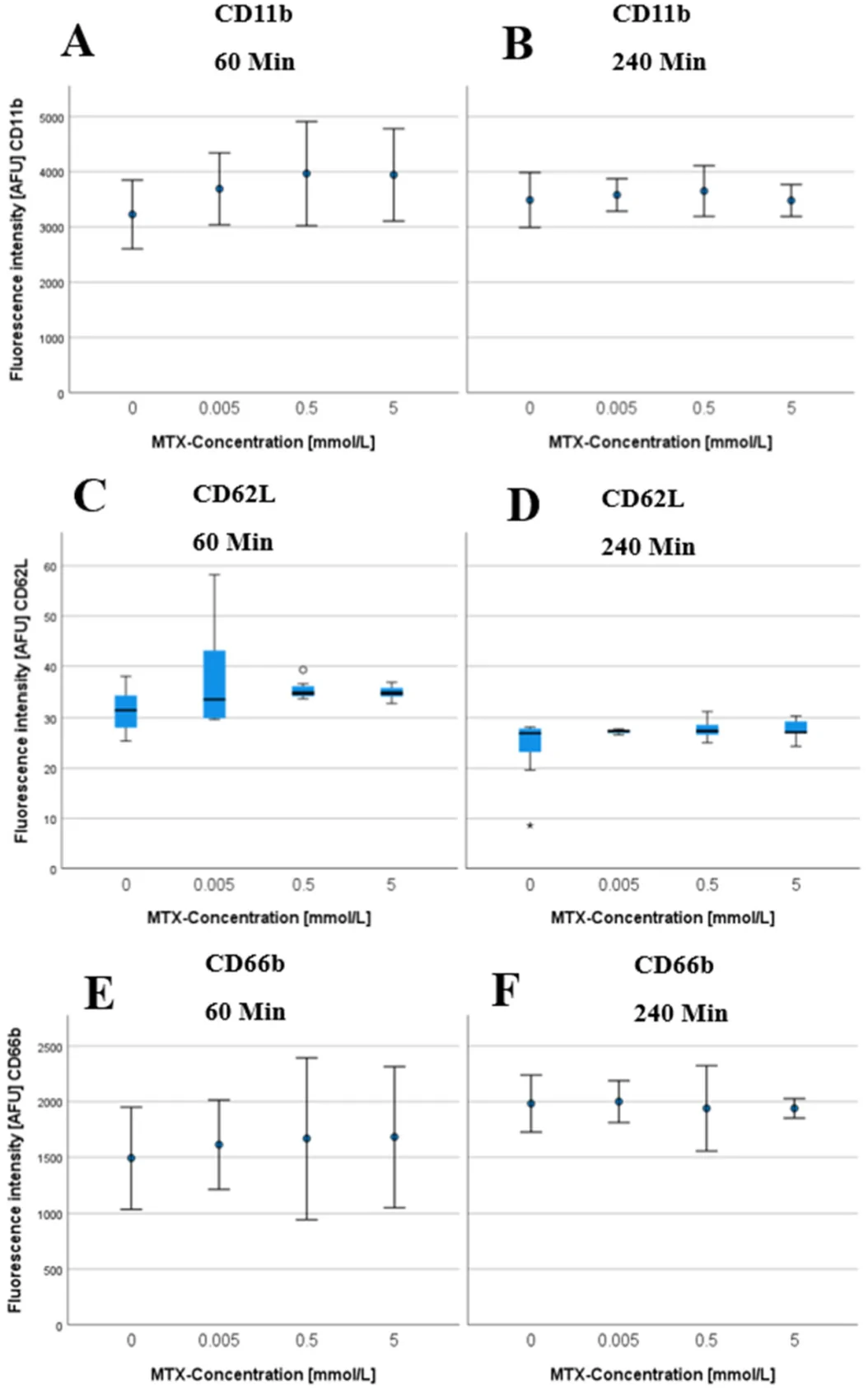
Results of flow cytometric analysis of the activation markers CD11b at 60 min (A) and 240 min (B), CD62L at 60 min (C) and 240 min (D), and CD66b at 60 min (E) and 240 min (F) each measured with 0, 0.005, 0.5 and 5 mmol/L MTX.

Similarly, no significant differences in CD62L expression were observed following incubation with MTX (60 min: *p* = 0.123; 240 min: *p* = 0.737; Figure 8C,D).

CD66b likewise showed no significant differences between the groups (60 min: *p* = 0.945; 240 min: *p* = 0.979; Figure 8E,F).

Overall, these results indicate that MTX does not affect the expression of the activation markers CD11b, CD62L, and CD66b.

In contrast, the surface marker LOX‐1 (see Figure 9) showed a different pattern. After 60 min of incubation with MTX, significant differences in LOX‐1 surface expression were observed. The surface expression of LOX‐1 was higher at a concentration of 5 mmol/L (145.4 [73.9–217.0]) compared with 0.005 mmol/L (55.9 [47.0–64.8]) (*p* = 0.034) and 0.5 mmol/L (66.2 [45.8–86.5]) (*p* = 0.045). The values at 0 mmol/L (62.0 [39.3–84.7]) and 5 mmol/L did not reach statistical significance (*p* = 0.08).

**Figure 9:**
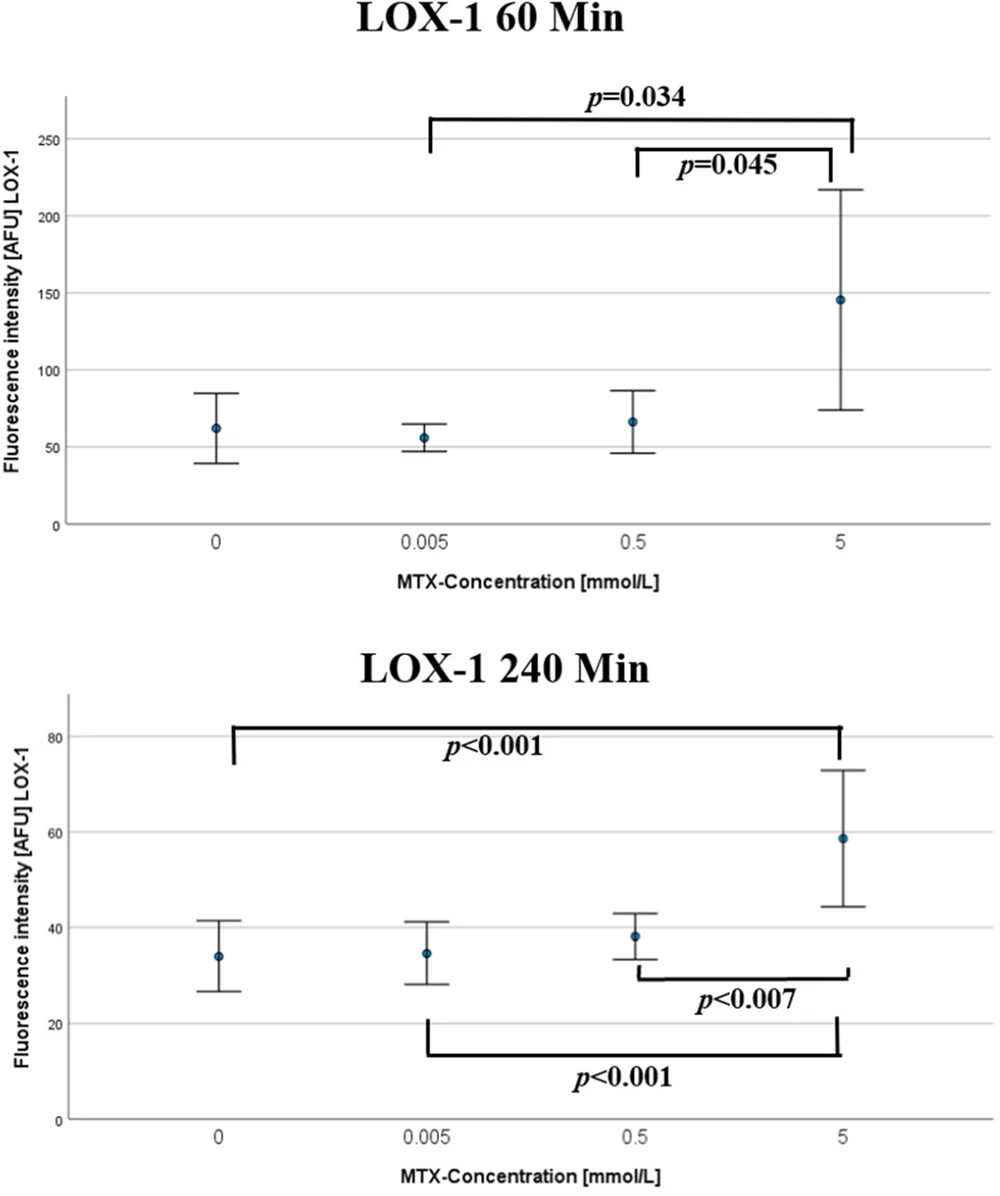
Comparison of mean values and 95% confidence intervals of fluorescence intensity for LOX‐1 expression as a function of MTX concentration (mmol/L) at 60 and 240 min.

At 240 min, the analyses again revealed significant differences. LOX‐1 expression at a concentration of 5 mmol/L MTX (58.7 [44.3–72.9]) was significantly higher than at 0 mmol/L (34.0 [26.7–41.4]) (*p* < 0.001), 0.005 mmol/L (34.7 [28.2–41.1]) (*p* < 0.001), and 0.5 mmol/L (*p* < 0.007) MTX (38.2 [33.4–42.9]).

#### 3.3.2 Analysis of the Respiratory Burst

No significant differences in fluorescence intensity were detected between MTX‐incubated and controls at 60 min (*p* = 0.737) or 240 min (*p* = 0.124) (see Figure 10).

**Figure 10:**
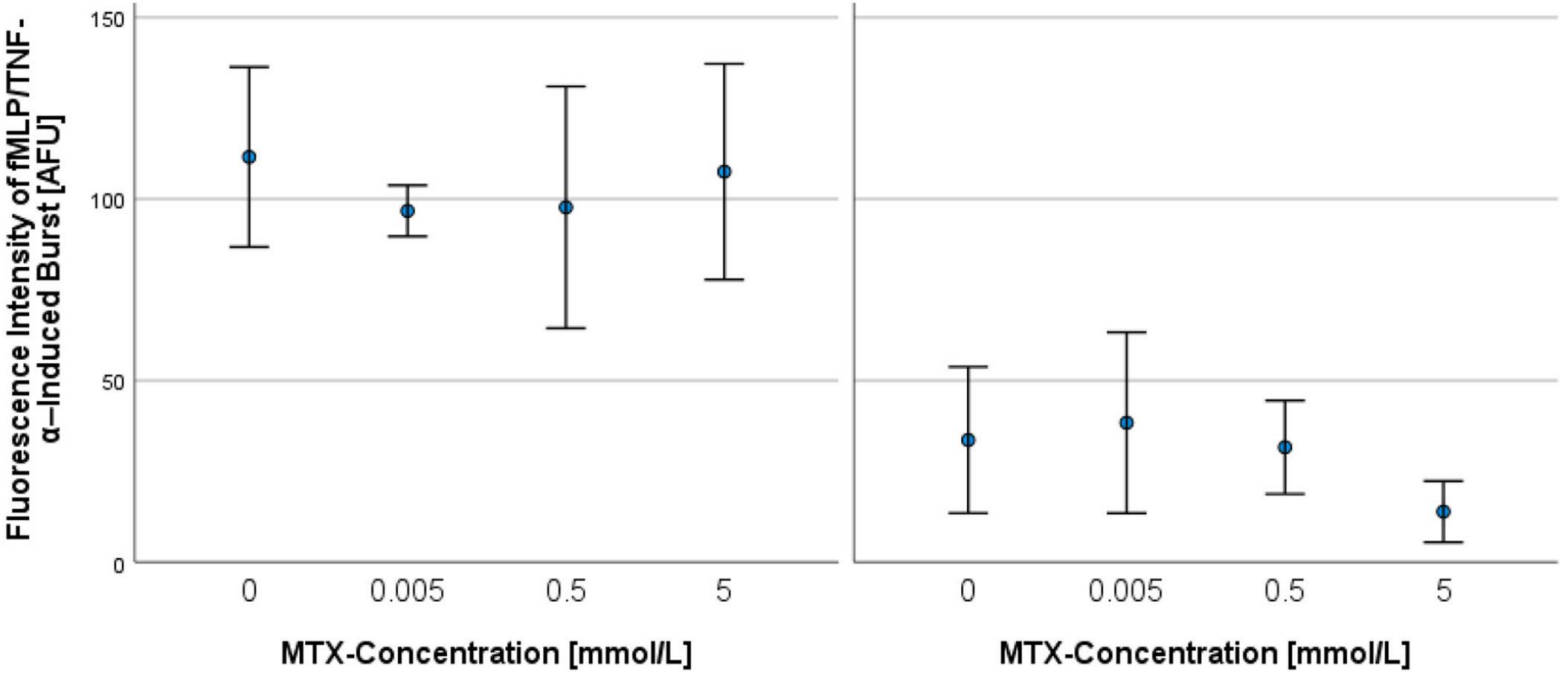
Comparison of mean fluorescence intensity (AFU) of the fMLP/TNF‐α–induced burst with 95% confidence intervals depending on MTX concentration (mmol/L) at 60 min (left) and 240 min (right).

A highly significant difference was observed when comparing the mean values across all MTX concentrations at 60 min (103.8) versus 240 min (29.6) (*p* < 0.001).

Table 2 shows the percentage of viable granulocytes.

**Table 2:** Percentage of viable granulocytes after incubation with 0, 0.005, 0.5, and 5 mmol/L MTX for 60 and 240 min.

MTX concentration [mmol/L]	Viable granulocytes	Viable granulocytes
	60 min [%]	240 min [%]
0	95.2	99.1
0.005	98.7	99.1
0.5	98.7	98.6
5	98.4	98.3

## 4 Discussion

PMNs play an important role in the pathogenesis of several autoimmune diseases. The aim of this study was to investigate how MTX affects key PMN functions. Central to our analysis were migration and alterations in surface epitopes, with particular focus on LOX‐1. To this end, a novel and gentle isolation method was applied in order to assess effects on PMNs in their most native state.

### 4.1 MTX Concentrations

Given the wide range of clinical indications for methotrexate, therapeutic plasma concentrations vary considerably depending on the treatment regimen. Whereas low‐dose MTX used for RA results in plasma concentrations within the micromolar range, high‐dose protocols for osteosarcoma and central nervous system malignancies can achieve peak concentrations exceeding 1.5 mmol/L [26, 27]. Therefore, MTX concentrations of 0.005 mmol/L and 0.5 mmol/L were chosen to cover clinically relevant exposure levels. A concentration of 5 mmol/L was additionally included to investigate potential effects at supratherapeutic concentrations.

### 4.2 Effect of MTX on Chemotaxis

For the chemokines fMLP and IL‐8, higher MTX concentrations were associated with a reduced chemotaxis of PMNs.

A particularly striking observation in the present experiments is that PMNs predominantly lose their chemotactic ability, while still retaining part of their ability to migrate. This difference is statistically significant (see Table 1 Supplement and Table 2 Supplement). It appears that MTX interferes with the cells’ ability to sense or respond to the chemoattractant gradient. Although PMNs continue to move—albeit with reduced total length compared to untreated controls—they are markedly less capable of directed migration toward the chemoattractant, as reflected by the significantly decreased TDX values. They frequently exhibit uncoordinated or non‐directional migration.

Several mechanisms may account for this effect. One hypothesis suggests that MTX inhibits migration through its folate antagonism at the DNA/RNA level, via inhibition of dihydrofolate reductase [16, 28]. However, this explanation appears unlikely, as the inhibitory effect on migration occurred within a very short time frame (already evident at half‐hour intervals 3 and 4; Figures 3 and 6). PMNs are terminally differentiated cells that are no longer capable of proliferation and exhibit a highly restricted transcriptional capacity. It is improbable that impaired nucleotide synthesis could result in such an immediate response, particularly given that PMNs, due to their short lifespan, rely only minimally on de novo synthesis [29, 30].

To exclude the possibility that this effect resulted from a specific interaction between MTX and the chemokine fMLP, additional experiments were performed using IL‐8 as the chemoattractant. These yielded comparable results (see Figure 6). The molecular mechanism underlying the reduced migratory activity and, in particular, the pronounced inhibition of chemotaxis remains unclear and warrants further investigation.

### 4.3 Effects of MTX on NETosis an ROS‐ Production

Microscopic analysis revealed no differences of neutrophil ROS formation or NETosis in the presence of MTX. The sensitivity of the method was confirmed using a positive control with PMA and TNF‐α. These findings are consistent with the flow cytometry results, which likewise showed no detectable ROS production.

MTX therefore does not induce cell death via NETosis, nor does it stimulate the formation of ROS. ROS production remained unchanged. This finding differs from the study by Kaundal et al. [31], who reported that PMNs from patients with RA receiving MTX produced less ROS than those from RA patients not treated with MTX. One possible explanation for this discrepancy is that, in patients with RA, not only MTX but also other additional factors may influence ROS production.

### 4.4 Effect of MTX on Surface Proteins

Flow cytometric analyses showed no upregulation of the markers CD11b and CD66b. These results (see Figure 8) indicate that MTX does not activate the cells, as the frequency of the aforementioned epitopes remains unchanged. This conclusion is further supported by the observation that l‐selectin (CD62L) also remains unaltered, a surface molecule typically downregulated upon activation [32].

Together, these three surface markers provide a coherent and internally consistent picture of the data.

Another surface molecule analyzed was LOX‐1. Our investigation clearly demonstrated that LOX‐1 is upregulated by MTX.

As shown in Figure 9, LOX‐1 expression was significantly upregulated following 5 mmol/L MTX administration, both after 60 and 240 min. LOX‐1 expression did not appear to be affected at concentrations of 0.05 mmol/L MTX and 0.5 mmol/L MTX.

As outlined in the introduction, LOX‐1 may serve as a potent biomarker for RA [19, 20]. The present study demonstrates that not only RA itself, but also MTX treatment can induce LOX‐1 expression. The extent to which these two factors interact and influence each other will need to be clarified in future studies.

### 4.5 Limitations

This study is limited by interindividual variability among PMN donors, which may have influenced the observed effects. Moreover, although the chemotaxis assays were designed to simulate physiological conditions, direct extrapolation of these *in vitro* findings to the *in vivo* human setting remains limited. Ultimately, centrifugation can impair PMNs, so we sought to minimize its use. Furthermore, this study exclusively investigated PMNs obtained from healthy donors. Therefore, the findings may not be directly transferable to patients with inflammatory diseases, such as RA or psoriasis, in whom PMN function and responses to MTX may differ. Additionally, the chemotaxis and flow cytometry data remain primarily descriptive. Further studies are therefore warranted to elucidate the underlying molecular mechanisms responsible for the observed effects.

## 5 Conclusion

The experiments conducted in this study clearly demonstrate that methotrexate influences the behavior of PMNs. MTX reduced the migratory capacity of these cells, particularly affecting chemotaxis, suggesting that PMNs lose their directional sensing ability. This effect appeared to be independent of the used chemoattractant. Furthermore, MTX increases the expression of LOX‐1, though the implications of this finding for the pharmacological action of MTX are not yet understood. Other surface markers (CD11b, CD62L, and CD66b), reactive oxygen species production, and NETosis were not affected by MTX. This indicates that MTX neither activates the cells nor drives them into cell death. The exact underlying molecular mechanisms remain to be elucidated, and provide promising potential for further research.

## Author Contributions

**Viktor Wieke:** conceptualization, methodology, validation, formal analysis, investigation, data curation, writing – original draft, visualization. **Richard Felix Kraus:** writing – review and editing, visualization. **Michael Andreas Gruber:** conceptualization, methodology, validation, formal analysis, resources, data curation, writing – review and editing, funding acquisition. **Martin Kieninger:** validation, data curation, writing – review and editing, supervision, project administration.

## Funding

The authors have nothing to report.

## Ethics Statement

The study was conducted in accordance with the Declaration of Helsinki (vote of the local ethics committee of the University of Regensburg [file number: 18‐1210‐101]).

## Consent

Written informed consent was obtained from all participants prior to sample collection.

## Conflicts of Interest

The authors declare no conflicts of interest.

## Supporting information


Supporting File

## Data Availability

The datasets used and/or analyzed during the current study are available from the corresponding author upon reasonable request.
