# Supplementary material for: Methotrexate Modulates Polymorphonuclear Neutrophil Function Through Reduced Chemotaxis and Increased LOX‐1 Expression
Source: Immun Inflamm Dis. 2026 Oct 6;14(10):e70537. doi: 10.1002/iid3.70537 (PMC13640531; doi:10.1002/iid3.70537)
Supplement: Supplementary file 1 [file IID3-14-e70537-s001.docx]

**Supplementary Material**

| **MTX concentration [mmol/L]** | **Median TL**  **[µm]** | **TL distance [%]** | **Median TDX** **[µm]** | **TDX distance [%]** |
| --- | --- | --- | --- | --- |
| 0 | 373.0 | 100.0 | 87.2 | 100.0 |
| 0.5 | 296.3 | 79.4 | 45.2 | 51.8 |
| 5 | 214.7 | 57.6 | 11.0 | 12.6 |

Table 1: Median values [µm] and relative decrease of TL and TDX across MTX concentrations using fMLP as a chemoattractant

| **MTX concentration [mmol/L]** | **Median TL**  **[µm]** | **TL distance [%]** | **Median TDX** **[µm]** | **TDX distance [%]** |
| --- | --- | --- | --- | --- |
| 0 | 350.7 | 100.0 | 69.5 | 100.0 |
| 0.5 | 326.0 | 93.0 | 50.8 | 73.1 |
| 5 | 237.7 | 67.8 | 10.3 | 14.8 |

Table 2: Median values [µm] and relative decrease of TL and TDX across MTX concentrations using IL-8 as a chemoattractant

| **Concentration [mmol/L]** | **CD11b (60 min) Mean [AFU] (95% CI)** | **CD11b (240 min) Mean [AFU] (95% CI)** | **CD62L (60 min) Median [AFU] [IQR]** | **CD62L (240 min) Median [AFU] [IQR]** | **CD66b (60 min) Mean [AFU] (95% CI)** | **CD66b (240 min) Mean [AFU] (95% CI)** |
| --- | --- | --- | --- | --- | --- | --- |
| 0 | 3226.4 (2602.6-3850.1) | 3487.7 (2988.3-3987.1) | 31.5 (7.38) | 27.0 (6.48) | 1492.6 (1035.7 – 1949.5) | 1982.1 (1726.4 – 2237.9) |
| 0.005 | 3690.2 (3039.5-4341.0) | 3579.9 (3285.0-3874.8) | 33,7 (15.0) | 27.4  (1.1) | 1612.9 (1211.8 – 2014.1) | 1999.8 (1811.3 – 2188.3) |
| 0.5 | 3966.2 (3023.1-4909.4) | 3650.4 (3190.5-4110.3) | 34,7  (3.2) | 27.4  (2.4) | 1668.0 (943.5 – 2392.6) | 1939.3 (1555.9 – 2322.7) |
| 5 | 3943.1 (3106.3-4779.9) | 3478.5 (3188.8-3768.2) | 34.8  (2.1) | 27.2  (3.6) | 1682.1 (1050.6-2313.7) | 1939.5 (1851.9-2027.1) |

Table 3: Expression of CD11b, CD62L, and CD66b (AFU) measured by flow cytometry after 60 and 240 min of MTX incubation

Figure 1: Representative graphs showing unstimulated PMNs, fMLP-stimulated PMNs, and PMA-stimulated PMNs (positive control). Axis labels: SSC-H: side scatter height; FSC-H: forward scatter height.
